# Polyethyleneimine Modified Two-Dimensional GO/MXene Composite Membranes with Enhanced Mg^2+^/Li^+^ Separation Performance for Salt Lake Brine

**DOI:** 10.3390/molecules29184326

**Published:** 2024-09-12

**Authors:** Jun Wang, Andong Wang, Jiayuan Liu, Qiang Niu, Yijia Zhang, Ping Liu, Chengwen Liu, Hongshan Wang, Xiangdong Zeng, Guangyong Zeng

**Affiliations:** 1 College of Biological and Chemical Engineering, Panzhihua University, Panzhihua 617000, China; enjoygreenlife@126.com (J.W.); pzh_niuqiang@163.com (Q.N.); 17764944212@163.com (Y.Z.); 15982680425@163.com (P.L.); 2 The 4th Geological Brigade of Sichuan, Chengdu 611130, China; pzhljy1990@163.com; 3 College of Materials and Chemistry & Chemical Engineering, Chengdu University of Technology, Chengdu 610059, China; chengwenliu@stu.cdut.edu.cn (C.L.); hongshanwang@stu.cdut.edu.cn (H.W.); zengxiandong17@cdut.edu.cn (X.Z.); 4 Tianfu Yongxing Laboratory, Chengdu 610213, China

**Keywords:** GO/MXene nanosheets, 2D nanosheet membranes, Mg^2+^/Li^+^ separation, salt lake brine, permeability and selectivity

## Abstract

As global demand for renewable energy and electric vehicles increases, the need for lithium has surged significantly. Extracting lithium from salt lake brine has become a cutting-edge technology in lithium resource production. In this study, two-dimensional (2D) GO/MXene composite membranes were fabricated using pressure-assisted filtration with a polyethyleneimine (PEI) coating, resulting in positively charged PEI-GO/MXene membranes. These innovative membranes, taking advantage of the synergistic effects of interlayer channel sieving and the Donnan effect, demonstrated excellent performance in Mg^2+^/Li^+^ separation with a mass ratio of 20 (Mg^2+^ rejection = 85.3%, Li^+^ rejection = 16.7%, S_Li,Mg_ = 5.7) in simulated saline lake brine. Testing on actual salt lake brine in Tibet, China, confirmed the composite membrane’s potential for effective Mg^2+^/Li^+^ separation. In the actual brine test with high concentration, Mg^2+^/Li^+^ after membrane separation is 2.2, which indicates that the membrane can significantly reduce the concentration of Mg^2+^ in the brine. Additionally, the PEI-GO/MXene composite membrane demonstrated strong anti-swelling properties and effective divalent ion rejection. This research presents an innovative approach to advance the development of 2D membranes for the selective removal of Mg^2+^ and Li^+^ from salt lake brine.

## 1. Introduction

Lithium, with its high specific heat capacity, allows it to efficiently absorb and release heat. Additionally, lithium’s low density contributes to the development of lighter and more efficient batteries. Furthermore, lithium exhibits high electrochemical activity and good ductility [1], making it widely applicable in aerospace, new energy, and pharmaceutical industries [2,3]. The development and utilization of lithium resources are crucial to the global energy transition. Salt Lake brine contains 80% of China’s lithium resources and is characterized by a relatively high mass ratio of Mg^2+^/Li^+^ [4,5,6]. The application of new materials and adsorbents makes the lithium extraction process more efficient while reducing the environmental impact. Global trends indicate that with the increasing demand for electric vehicle batteries, brine lithium extraction technologies are rapidly evolving towards greater efficiency and environmental sustainability. Belonging to the diagonal elements, the physical and chemical properties of lithium and magnesium are quite similar [7]. Thus, the separation of Mg^2+^/Li^+^ in salt lake brine poses significant challenges for lithium extraction [8]. Common lithium extraction technologies include extraction, electrodialysis, adsorption, and precipitation [9,10,11,12]. However, these methods are hindered by severe equipment corrosion, high consumption of chemical reagents, and significant power requirements [13]. Therefore, developing a saline lithium extraction technology with high Mg^2+^/Li^+^ separation efficiency, energy efficiency, and environmental sustainability is essential.

In recent years, membrane separation technology has become a popular technology in desalination, food processing, and wastewater purification due to its high separation performance, low chemical reagent consumption, and no phase change [14,15]. The 2D membranes constructed with 2D materials have attracted increasing research interest owing to their unique interlayer permeation channels and high separation efficiency [16]. Commonly used 2D materials include graphene oxide (GO), transition metal carbides/nitrides (MXene), graphitic carbon nitride, and transition metal sulfides [17,18]. Among them, GO has the advantages of excellent hydrophilicity, abundant active functional groups (carboxyl, hydroxyl, carbonyl), and a large specific surface area [19,20,21]. Zhao et al. [22] used vacuum filtration to combine 0D GO quantum dots (QDs) with 2D GO nanosheets, successfully fabricating GO QDs/GO membranes with high permeability. The incorporation of GO QDs effectively enhanced the hydrophilicity and interlayer spacing of the membranes. Experimental results demonstrated that the membranes achieved excellent dye and macromolecular protein removal efficiencies, consistently maintaining removal rates above 99%. Moreover, the membranes exhibited permeation performance that was two to four times higher than that of pristine GO membranes. However, the study still did not solve the problem of poor anti-fouling ability of the membranes. Zhan et al. [23] precisely regulated the interlayer channels of GO membranes, overcoming the trade-off effect and achieving outstanding desalination ability. POSS@GO hybrid membranes were fabricated by compositing GO nanosheets with aminopropyl isobutyl polyhedral oligomeric silsesquioxane (NH_2_-POSS). Glutaraldehyde was used as the crosslinking agent. In order to improve the permeability and desalination performance of the composite membranes (water flux = 112.7 kg/(m^2^·h), rejection of NaCl > 99%), the polyhedral structure of POSS could effectively increase the interlayer spacing of layered GO, providing a suitable spatial hindrance effect. However, due to its excellent hydrophilicity, GO membranes readily form hydrogen bonds with water molecules in aqueous solutions, leading to swelling phenomena.

MXene is a family of 2D transition metal carbides, nitrides, or carbonitrides that can be synthesized by etching the precursor M_n+1_AX_n_ phase [24,25]. M is the element of the transition metal, A is usually the element Al, and X is the element of carbon or nitrogen. The structural formula of MXene is generally written as M_n+1_X_n_T_x_, where T_x_ represents the −O-O, −OH, and −F groups formed during etching [26,27]. These functional groups endow MXene nanosheets with good chemical modification and hydrophilicity [28]. Wang et al. [29] intercalated MXene as a guest material into rGO membranes, significantly improving their hydrophilicity. The 2D/2D rGO/MXene permeation channels were constructed, and the composite membrane demonstrated outstanding performance, with a pure water flux of 62.1 L·m^−2^·h^−1^·bar^−1^ and 91.4% methyl orange rejection. However, the study did not address the long-term stability of the membrane, a critical parameter for assessing membrane performance. Mg^2+^ and Li^+^ have similar ionic radii (4.3 A and 3.8 A, respectively), making selective separation challenging when relying solely on the sieving effect of layer spacing [30]. The surface charge properties of 2D membranes significantly influence the electrostatic repulsion of Mg^2+^ and Li^+^ ions, as explained by the Donnan effect [31,32]. Positively charged membrane surfaces have different electrostatic repulsive forces for Mg^2+^ and Li^+^. This results in differing mass transfer rates, thereby enabling the selective separation of Mg^2+^ and Li^+^ ions [33,34].

In this study, a simple pressure-assisted filtration and surface coating method to prepare PEI-GO/MXene composite membranes with a 2D/2D structure and positive charge. Firstly, high-quality MXene nanosheets were synthesized through etching with LiF and HCl. Subsequently, GO and MXene were homogeneously dispersed and pump-filtered onto polyethersulfone (PES) substrate membranes. The surface of the composite membrane was treated with a polyethyleneimine (PEI) coating via an electrostatic attraction process. This process altered the surface charge characteristics of the composite membrane. Furthermore, the effects of different PEI coating conditions on membrane separation performance were investigated. The composite membrane’s Mg^2+^/Li^+^ separation performance was also evaluated. In this study, the intercalation of MXene into the GO layer exhibited enhanced stability, which was conducive to the long-term use of the membrane. Compared with most membranes used for magnesium-lithium separation, PEI-GO/MXene membranes significantly reduced Li^+^ retention due to the Donnan effect.

## 2. Results and Discussion

### 2.1. Characterization of Materials and Composite Membranes

The microscopic structures of GO and MXene nanomaterials were examined using scanning electron microscopy (SEM). GO nanosheets exhibited a typical 2D layered structure, as depicted in Figure S1a. The interlayer bonding was relatively tight, and the lateral size of the GO nanosheets was comparatively large. The MAX phase displayed a dense, blocky structure and was characterized by a tightly packed arrangement with minimal gaps between layers (Figure S1b). In contrast, MXene nanomaterials exhibited increased interlayer spacing following etching and ultrasonic stripping (Figure S1c). The interlayer spacing increased, and the lateral dimensions of the sheets were about a few micrometers. This loose interlayer structure makes MXene highly suitable for two-dimensional membrane construction.

**Figure 1:**
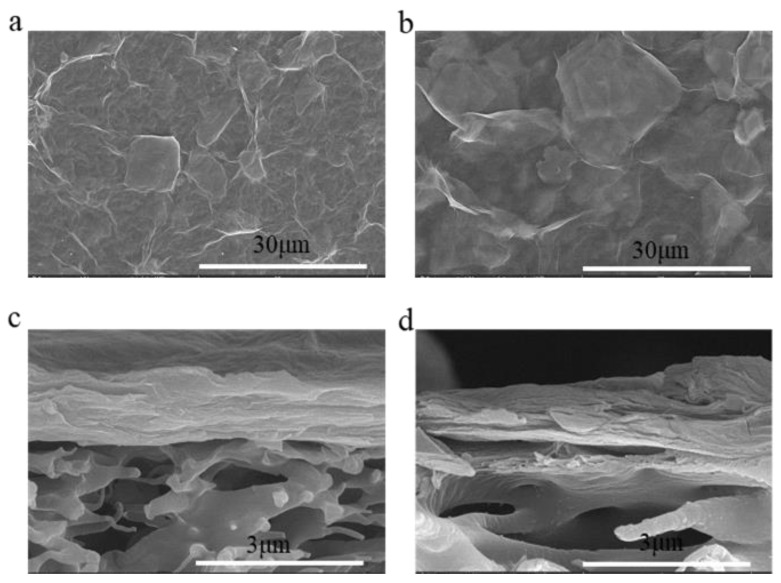
(**a**,**b**) SEM images showing the surfaces of M0 and M3; (**c**,**d**) SEM images of the cross-sections of M0 and M3.

As illustrated in Figure 1, SEM images of the M0 and M3 membranes are presented. A distinctive folded structure was observed on the surface of the M0 composite membrane (Figure 1a), which is a characteristic morphology of GO-based double-layered 2D membranes [35]. Furthermore, the observed flakes were larger in size and identified as GO-related. However, the overlay of larger GO nanosheets on smaller MXene nanosheets obscured these features, making them less apparent on the composite membrane’s surface. In contrast, the surface folds of the M3 membrane were significantly reduced, resulting in a markedly smoother appearance (Figure 1b). This phenomenon was attributed to hydrogen bonding and electrostatic attractions between the amine groups of PEI and the oxygen-containing functional groups of GO and MXene. These interactions likely contribute to the formation of a dense organic layer on the membrane surface [36]. The dense organic layer enhances Mg^2+^ retention by the membrane. Additionally, Figure 1c illustrates that the cross-section of the M0 composite membrane exhibited a typical lamellar structure with 2D nano-channels capable of intercepting larger substances and facilitating the transport of small targets and water molecules [37]. After PEI surface coating (Figure 1d), the cross-section of the composite membrane showed no obvious changes, and the thickness of the laminate structure remained nearly unchanged. This suggests that the PEI surface coating had minimal impact on the membrane’s cross-sectional structure.

**Figure 2:**
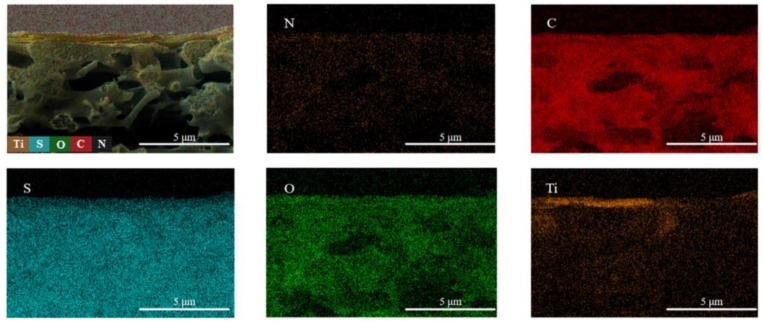
The EDS-mapping of M3.

Elemental distribution in the composite membrane was characterized using EDS mapping on M3, as shown in Figure 2. C, N, and O elements were uniformly distributed throughout the PES base membrane and its laminar structure. S element was primarily distributed in the PES membrane, while the aggregation of the Ti element was more obvious at the top of the membrane. This distribution pattern is attributed to the uniform dispersion of MXene nanosheets within the separated layers of the composite membrane, which prevents agglomerate formation.

**Figure 3:**
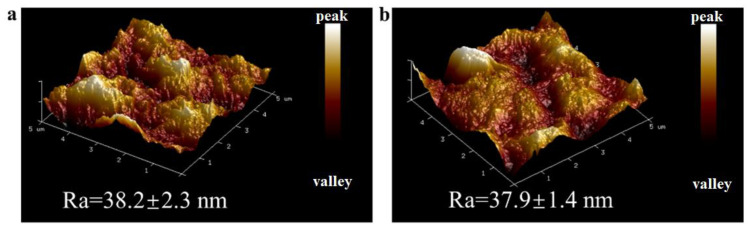
(**a**,**b**) Images of the M0 and M3 samples obtained by AFM.

Atomic force microscopy (AFM) analysis was performed on two composite membranes, as shown in Figure 3. Before modification, the surface roughness of the composite membrane was 38.2 ± 2.3 nm, which slightly decreased to 37.9 ± 1.4 nm after modification. The coating did not significantly impact the surface roughness of the membrane. The considerable surface roughness prior to modification contributed to the enhanced permeability of the composite membrane [38].

**Figure 4:**
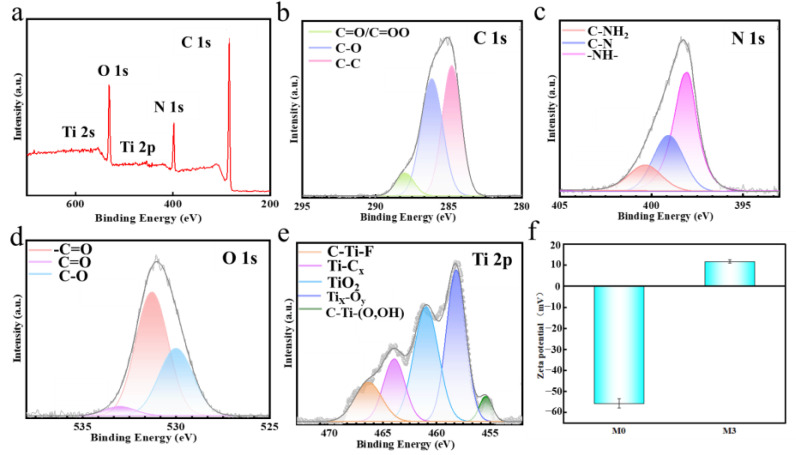
(**a**) Full spectra of XPS for M3; (**b**–**e**) The high-resolution spectra of the carbon 1s, nitrogen 1s, oxygen 1s, and titanium 2p electrons in M3; (**f**) Zeta potential of M0 and M3.

Figure 4 presents the XPS energy spectrum of the M3 composite membrane, with Table 1 providing the elemental composition. Significant characteristic peaks at 284.8 eV (C 1s), 399.3 eV (N 1s), 531.5 eV (O 1s), and 457 eV (Ti 2p) were observed in the full spectrum (Figure 4a) [39,40,41]. The presence of numerous amine groups in PEI produced a more pronounced N 1s peak in the full spectrum of the composite membrane, confirming successful PEI incorporation. The O 1s peaks originated from oxygen-containing groups on the surface of GO and MXene. C 1s peaks indicated the presence of carbon, mainly from GO, MXene, and PEI. Ti was derived from MXene, confirming its successful intercalation into GO. The C 1s spectrum (Figure 4b) revealed the presence of C–C/C=C, C–O, and C=O/COOH functional groups in GO nanosheets, with peaks at 284.8 eV, 286.9 eV, and 288.5 eV, respectively. In addition, peaks at 401 eV (C–NH_2_) and 399 eV (C–N) were detected in the N1s spectrum (Figure 4c). The O 1s spectrum (Figure 4d) showed a fitted peak corresponding to N–C=O. This peak resulted from nitrogen in the amine group of the PEI molecule [36]. Peaks at 455.8 eV (C–Ti–(O, OH)), 458.7 eV (Tix–Oy), 460.2 eV (TiO_2_), 461.6 eV (Ti–Cx), and 464.4 eV (C–Ti–F) were observed, as shown in Figure 4e. These results confirm the successful preparation of PEI-GO/MXene composite membranes. According to the data in Figure 4f, the zeta potential of the PEI unmodified composite membrane was −41.9 mV. This negative potential is attributed to oxygen-containing functional groups on the surfaces of GO and MXene nanosheets, such as carboxyl (–COOH), hydroxyl (–OH), and oxygen (–O). PEI amine groups interacted and adhered to the membrane surface upon modification. Protonation of amine groups in water generated a positive potential (+11.8 mV) on the M3 membrane surface [28]. According to the Donnan effect, positively charged membrane surfaces exhibit greater electrostatic repulsion towards cations. Consequently, the introduction of PEI improved Mg^2+^ rejection in the PEI-GO/MXene membrane, enhancing its Mg^2+^/Li^+^ separation capacity.

**Figure 5:**
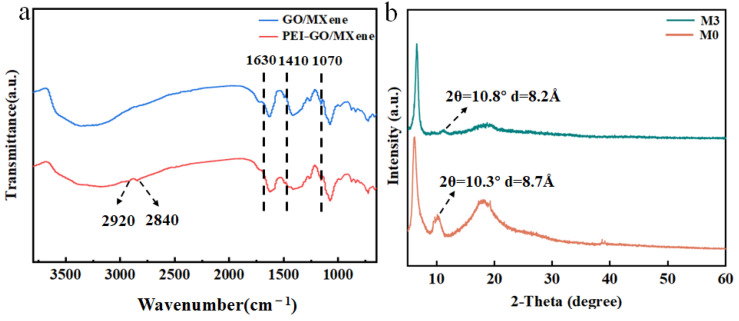
The FTIR (**a**) and XRD (**b**) patterns of M0 and M3.

Figure 5a illustrates the Fourier-transform infrared spectroscopic (FTIR) results for the composite membrane. A notable peak at 1630 cm^−1^ corresponds to the stretching vibration of the carbonyl group, specifically the carboxyl group (C=O). This peak is attributed to the widespread presence of the oxygen-containing functional group –COOH in GO nanosheets [42]. The distinctive peak at 1410 cm^−1^ indicates the carbon-carbon out-of-plane stretching vibration on the sp^2^ carbon skeleton. The peak at 1070 cm^−1^ is attributed to the stretching vibration of the C–O bond, originating from GO or MXene nanosheets. In the PEI-GO/MXene membrane, peaks at 2920 cm^−1^ and 2840 cm^−1^ correspond to the stretching vibrations of the amine group. This is due to abundant amine functional groups in PEI. These results suggest the successful incorporation of PEI into the composite membranes [36].

**Figure 6:**
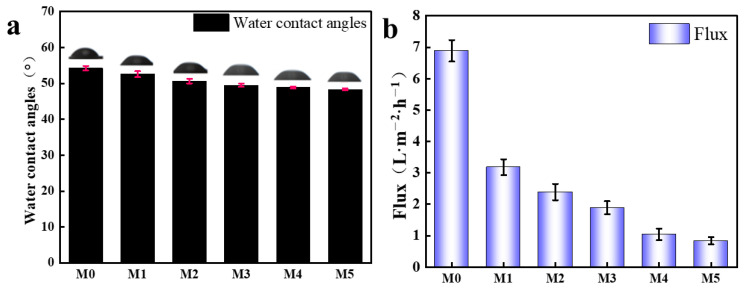
(**a**) The water contact angles and (**b**) the pure water flux of various composite membranes.

Figure 5b displays the X-ray diffractometry (XRD) patterns of the M0 and M3 composite membranes. The GO characteristic peak in M0 corresponds to 2θ = 10.3, and according to the Bragg equation, the interlayer spacing of the M0 composite membrane was calculated to be 8.7 Å [43]. According to the literature [44], the diameter of hydrated Mg^2+^ (~8.56 Å) was slightly smaller than the interlayer spacing of M0. Furthermore, the Donnan repulsion of cations on the negatively charged surface of M0 is weak. Therefore, M0 could not achieve effective Mg^2+^ rejection through the combination of size sieving and the Donnan effect. For M3, the characteristic peak shifted to 10.8°, corresponding to an interlayer spacing of 8.2 Å. The formation of a polymer layer on the PEI membrane surface reduced the interlayer spacing of M3. As a consequence of the reduced layer spacing, the permeability coefficient of water molecules increases, while the overall permeability performance of the composite membrane decreases [28]. However, this reduction enhances the ion rejection capability of M3. As the PEI concentration in the composite membrane increases, the water contact angle gradually decreases (Figure 6a). With increasing PEI concentration, the number of amine groups on the membrane surface increases. This increase leads to the formation of additional hydrogen bonds, strengthening the interaction between the membrane surface and water molecules [37]. The dense PEI network structure reduces membrane flux and increases the Mg^2+^ retention rate.

### 2.2. Performance Testing of Composite Membranes

Water flux variation at 2 bar pressure for different composite membranes is shown in Figure 6b. The water flux of the M0 composite membrane reached 6.9 L·m^−2^·h^−1^. However, as the PEI concentration increased, the water flux decreased to 1.98 L·m^−2^·h^−1^ at a PEI concentration of 1.5 wt%. Generally, PEI modification made the composite membrane more hydrophilic. PEI enhances the adhesion of water molecules to the surface, improving water flow through the material [45]. Nevertheless, PEI interacts with oxygen-containing functional groups on the surface of GO/MXene composite membranes through hydrogen bonding and electrostatic interactions, resulting in the formation of a dense membrane and reduced water flux [31].

**Figure 7:**
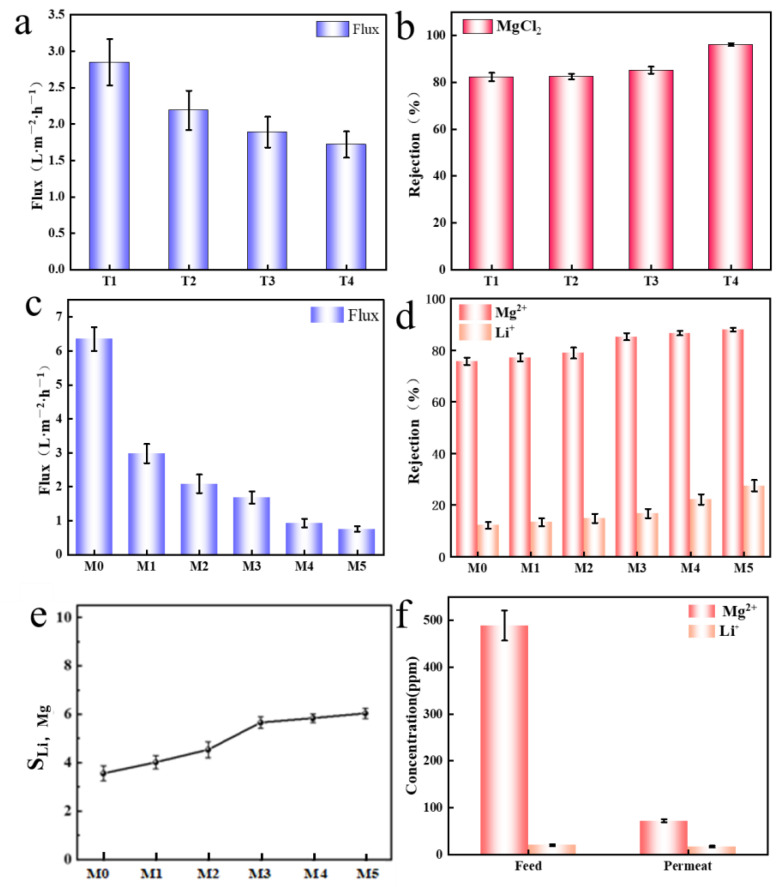
(**a**,**b**) Flux and Mg^2+^ rejection of composite membranes at different coating times; (**c**) Flux and (**d**) Mg^2+^ and Li^+^ rejection for simulated salt lake brine; (**e**) S_Li,Mg_ for different composite membranes; (**f**) Separation capacity of M3 for Mg^2+^ and Li^+^.

Additionally, the effect of coating time on composite membrane performance was evaluated (T = 5 min, 15 min, 30 min, 60 min), and the separation efficiency was investigated using a 1 g/L MgCl_2_ solution. The composite membrane flux gradually decreased with increasing coating time (Figure 7a). This was due to the formation of thicker polymer layers over time, leading to higher resistance to water molecule permeation and reduced permeability performance. The rejection rate of the composite membrane for MgCl_2_ gradually increased with longer PEI coating times. The final MgCl_2_ rejection was 86.2% at the time of T4 (Figure 7b). This represents an improvement of over 20% compared to the 65.2% MgCl_2_ rejection of the GO/MXene membrane. Therefore, the optimal PEI concentration was determined to be 1 wt%, with a coating time of 30 min.

Simulated salt lake brine (1866 ppm MgCl_2_ and 134 ppm LiCl) was used to evaluate the Mg^2+^/Li^+^ separation performance of various composite membranes. The flux gradually decreased with increasing PEI concentration, which was similar to the decreasing trend of water flux (Figure 7c). Furthermore, the Mg^2+^ rejection rates of the composite membranes were 70.68%, 75.76%, 85.27%, 86.66%, and 87.99%, respectively (Figure 7d). MgCl_2_ rejection increased rapidly before the PEI concentration of 1 wt%. When PEI concentrations were greater than 1 wt%, the increase in MgCl_2_ rejection was not significant. The increased membrane surface charge at higher PEI concentrations enhanced electrostatic repulsion, improving S_Li,Mg_ (Figure 7e). After further increasing the PEI concentration, there was no significant improvement in the Mg^2+^/Li^+^ selectivity of the composite membrane, so M3 was considered the best membrane (S_Li,Mg_ = 5.7). The brine contained 489.4 ppm Mg^2+^ and 20.3 ppm Li^+^, as illustrated in Figure 7f. Following treatment with M3, the permeate contained 58.8 ppm Mg^2+^ and 14.7 ppm Li^+^. Combining interlayer sieving with electrostatic repulsion [46], M3 achieved 85.27% rejection of MgCl_2_ and only 16.68% rejection of LiCl. The composite membrane exhibited a strong affinity forMg^2+^, resulting in significant retention. It demonstrated low Li^+^ rejection, resulting in effective Mg^2+^/Li^+^ separation [47,48,49].

**Figure 8:**
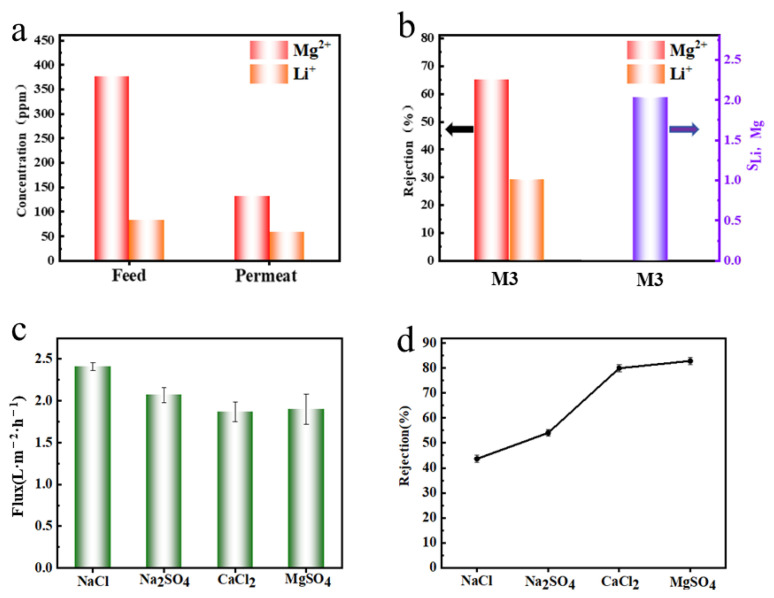
(**a**) Mg^2+^ and Li^+^ concentration before and after filtration; (**b**) Effectiveness of M3 in selectively separating Mg^2+^ and Li^+^ from diluted brine solutions; (**c**,**d**) Flux and rejection of NaCl, Na_2_SO_4_, CaCl_2_ and MgSO_4_ solutions by M3 composite membrane.

Due to the significant differences between the compositions of actual and simulated salt lake brines, this study further investigated the Mg^2+^/Li^+^ separation efficiency of composite membranes on real saltwater brines. Actual brine samples were sourced from a region of Tibet, China, and underwent flotation, filtration, and other pre-treatments, with specific compositions detailed in Table S2. The salt lake brine was diluted tenfold before use. The feed solution contained 377 ppm of Mg^2+^ and 84 ppm of Li^+^, resulting in a Mg^2+^/Li^+^ ratio of 4.5. The concentrations of Mg^2+^ and Li^+^ in the permeation solution after treatment with the composite membrane were 132 and 59 ppm, respectively, with Mg^2+^/Li^+^ = 2.2 (Figure 8a). These results demonstrated that the M3 significantly reduced the concentration of Mg^2+^ in the salt lake brine, thereby achieving the desired reduction in the Mg^2+^/Li^+^ mass ratio. As shown in Figure 8b, the Mg^2+^ rejection rate of M3 was 64.8%, and the Li^+^ rejection rate was 29.8%, with S_Li,Mg_ = 2. Compared to the simulated brine, the composite membrane’s Mg^2+^/Li^+^ separation efficiency was decreased. First, the total salinity of the salt lake brine remained high even after dilution, intensifying the effect of concentration polarization. In addition, high salinity shielded some of the membrane charges and attenuated the Donnan effect, leading to a reduction in Mg^2+^ rejection. The Donnan effect imparted selectivity to the membrane for ions with different charges, which was particularly significant in this study. The Donnan effect also influenced the distribution of ions on both sides of the membrane, contributing to ion balance. Since the membrane carried a fixed internal charge, ion distribution on both sides had to maintain electrical neutrality, leading to a lower concentration of Mg^2+^ relative to Li^+^ inside the membrane and enhancing the selective transmission of Li^+^. PEI modification increased the membrane’s positive charge, making it more selective for Li^+^ passage while rejecting Mg^2+^, thus achieving effective Mg^2+^/Li^+^ separation [50]. Additionally, the salt lake brine contained monovalent Na^+^ and K^+^, which competed with Li^+^ for permeation and potentially hindered Mg^2+^/Li^+^ separation [51].

As illustrated in Figure 8c,d, the separation performance of the composite membrane was evaluated using 1 g/L of CaCl_2_, MgSO_4_, Na_2_SO_4_, and NaCl solutions. The flux order was NaCl > Na_2_SO_4_ > CaCl_2_ > MgSO_4_, while the salt rejection order was MgSO_4_ (94.8%) > CaCl_2_ (84.1%) > Na_2_SO_4_ (81.4%) > NaCl (36.9%). The PEI coating created a positively charged membrane surface, resulting in stronger electrostatic repulsion for divalent ions Mg^2+^ and Ca^2+^. This decreased the permeability of the salt solutions but increased rejection efficiency. Due to the smaller charge and radius of Na^+^ and the smaller hydration radius of Cl^−^ compared to SO_4_^2−^, NaCl rejection was lower than that of Na_2_SO_4_ [52,53].

To test its swelling resistance, M3 was immersed in a beaker containing 50 mL of salt lake brine. The immersion times of the composite membrane were 24 h, 72 h, and 168 h. As shown in Figure S2a–c, the surface of M3 did not exhibit any peeling or color change after different immersion times. This indicates that the composite membrane demonstrated excellent resistance to swelling in salt lake brine, maintaining its stability and integrity throughout the immersion period. Furthermore, even when bent to 90° (Figure S2d), M3 remained intact without peeling.

## 3. Experiments

### 3.1. The Synthesis of MXene Nanosheets

The synthesis of MXene nanosheets was achieved through a two-step process involving the etching of LiF + HCl mixed solutions and ultrasound-assisted stripping [54,55]. The detailed preparation procedure is described in Supplementary Methods S2. Additionally, Supplementary Methods S1 lists the materials used, and Supplementary Methods S3 outlines the characterization conditions.

### 3.2. The Fabrication of a PEI-GO/MXene Composite Membrane

**Figure 9:**
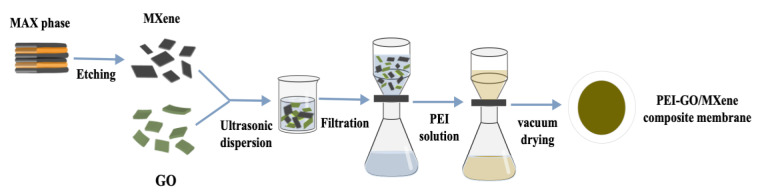
The schematic diagram depicts the methodology employed in the fabrication of the PEI-GO/MXene composite membrane.

Initially, a specific quantity of GO and MXene powder was weighed and added to a glass vial containing deionized water, respectively, and ultrasonically dispersed for 2 h at a concentration of 0.5 mg/mL in an ice bath. The GO and MXene dispersions were then transferred into two beakers containing deionized water and ultrasonically dispersed for 15 min. The two dispersions were combined and sonicated for 30 min to create a homogeneous GO/MXene dispersion. The dispersion was filtered through a dead-end filtration device (Figure S3) with a PES-based membrane, followed by soaking in 20 mL of PEI solution at varying concentrations for a certain time. After coating, the PEI solution was removed, and the membrane surface was rinsed with deionized water. The membrane was then vacuum-dried in an oven at 40 °C for 1 h, resulting in the formation of a PEI-GO/MXene composite membrane, as illustrated in Figure 9. The ratios and numbers of different composite membranes are presented in Table S1.

## 4. Conclusions

This study employed PEI for surface coating modification to fabricate positively charged PEI-GO/MXene composite membranes. The preparation, characterization, and Mg^2+^/Li^+^ separation performance of the composite membranes were analyzed to elucidate the membrane-forming mechanism and the constitutive relationships of these new composite membranes. The findings indicated that MgCl_2_ rejection by the composite membrane progressively improved with increasing PEI concentrations, while the water flux decreased correspondingly. When the coating time T3 was 30 min and PEI concentration was 1 wt%, the M3 composite membrane achieved a MgCl_2_ rejection rate of 85.27%, a LiCl rejection rate of 16.68%, and the S_Li,Mg_ value as high as 5.7, realizing efficient Mg^2+^/Li^+^ separation. In addition, the composite membrane demonstrated good Mg^2+^/Li^+^ selectivity and resistance to swelling when tested with actual salt lake brine. The experimental results show that strengthening the Donnan effect is a very effective starting point for magnesia-lithium separation, which will enhance the magnesia-lithium separation performance of the membrane. This study is expected to provide experimental guidance for constructing high-performance 2D composite membrane materials based on GO and offer innovative approaches for developing novel membrane materials for lithium extraction from salt lake brine. Although the membrane exhibits good magnesia-lithium separation performance, the narrow channels of the GO membrane limit its water flux. In future research, increasing the flux of the membrane is an urgent problem to be solved.

## Figures and Tables

**Table 1:** Elemental composition of M0 and M3 based on XPS analysis.

Type of Membrane	C (%)	O (%)	Ti (%)	N (%)
M3	70.81	15.87	0.15	13.16

## Data Availability

The data that supports the findings of this study is available.

## Supplementary Materials

The following supporting information can be downloaded at: https://www.mdpi.com/article/10.3390/molecules29184326/s1, Figure S1: (a–c) SEM images of GO nanosheets, MAX phase, and MXene nanosheets; Figure S2: Digital images of M3 after different immersion times in salt lake brine: (a) 24 h, (b) 72 h, (c) 168 h; (d) bending picture of M3; Figure S3: The dead-end filtration device; Table S1: Composition of different composite membranes. Table S2: Actual salt lake brine ion concentration in Tibet, China.
