# Supplementary material for: Polyethyleneimine Modified Two-Dimensional GO/MXene Composite Membranes with Enhanced Mg2+/Li+ Separation Performance for Salt Lake Brine
Source: Molecules. 2024 Sep 12;29(18):4326. doi: 10.3390/molecules29184326 (PMC11433666; doi:10.3390/molecules29184326)
Supplement: Supplementary file 1 [file molecules-29-04326-s001.zip › molecules-3165974-supplementary.pdf]

Supplementary Materials

Polyethyleneimine modified two-dimensional GO/MXene composite membranes with enhanced Mg^{2+}/Li^{+} separation performance for salt lake brine

Jun Wang [1], Andong Wang [2, *], Jiayuan Liu [2], Qiang Niu [1], Yijia Zhang [1], Ping Liu [1], Chengwen Liu [3], Hongshan Wang [3], Xiangdong Zeng [3] and Guangyong Zeng [3, 4, *]

1. College of Biological and Chemical Engineering (College of Agricultural Sciences), Panzhihua University, Panzhihua, Sichuan 617000, PR China; enjoygreenlife@126.com (J.W.); pzh_niuqiang@163.com (Q.N.); 17764944212@163.com (Y.Z.); 15982680425@163.com (P.L.)
2. The 4th Geological Brigade of Sichuan, Chengdu, Sichuan 611130, PR China; PZHLJY1990@163.com (J.L.)
3. College of Materials and Chemistry & Chemical Engineering, Chengdu University of Technology, Chengdu, Sichuan 610059, PR China; chengwenliu@stu.cdut.edu.cn (C.L.); hongshanwang@stu.cdut.edu.cn (H.W.) zengxiandong17@cdut.edu.cn (X.Z.)
4.Tianfu Yongxing Laboratory, Chengdu, Sichuan 610213, PR China

* Correspondence: Email: 13882330145@163.com (A.W); wuwu5125@163.com (G.Z); Phone and Fax: +86(0)28-8407-3864 (G.Z)

1. Supplementary Methods

S1. Materials

PES microfiltration membranes with a diameter of 50 mm and a pore size of 0.22 μm were obtained from Haining Yibo (Zhejiang, China). MAX phase was purchased from 11 Technology Co., Ltd. (Jilin, China). HCl and PEI (Mw=70,000) were supplied by Aladdin Reagent Co. NaCl, LiCl, LiF, and $MgSO_4$ were sourced from Kelong Chemical Co., Ltd (Chengdu, China). $CaCl_2$, Na_2SO_4, and $MgCl_2$ were provided by McLean Co., Ltd. (Shanghai, China).

S2. Synthesis of MXene nanosheets

First, 1.5 g of LiF was weighed and dissolved in 40 ml of 9 M HCl. Then, 2 g of MAX phase was added to the solution, and the mixture was magnetically stirred for 20 hours at 25℃. After etching, the mixture was centrifuged at 6000 rpm and washed several times with deionized water. The resulting precipitation was then dispersed into 50 mL of distilled water and ultrasonic treated in a nitrogen atmosphere for 8 hours. The resulting supernatant was collected and finally freeze-dried to obtain monolayer or few-layer MXene nanosheets.

S3. Characterization

The morphology and structure of the nanomaterials and composite membranes were observed using scanning electron microscopy (SEM). X-ray diffraction (XRD) was employed to test the interlayer spacing of the composite membranes. Atomic force microscopy (AFM) was utilized to characterize the surface roughness of the composite membranes. Fourier-transform infrared spectroscopy (FTIR) and X-ray photoelectron spectroscopy (XPS) were employed to analyze the changes of functional groups and elemental compositions on the surface of the composite membranes. Furthermore, the surface hydrophilicity of the composite membranes was measured using a contact angle tester. Use a zeta potential analyzer to characterize the surface charge properties of the composite membranes.

S4. Membrane performance test

In this study, a dead-end filtration device was employed to examine the pure water flux of composite membranes. Each membrane was pre-pressurized at a pressure of 2.5 bar for 0.5 h, followed by maintaining a pressure of 2 bar, and the permeate deionized water volume was recorded and collected every 0.5 h. Each membrane was tested three times,

and the average value was taken as the pure water flux. The pure water flux of the composite membrane was calculated according to equation S1:

$$J=\frac{V}{A\times t} \quad \text{(S1)}$$

where, V is the water volume (L); A is the effective filtration area of the membrane (m^2); t is the permeation time (h).

The ion retention ability of the composite membrane for different salt solutions (NaCl, Na_2SO_4, LiCl, $MgCl_2$, $MgSO_4$, $CaCl_2$, 1 g/L) was evaluated by the rejection rate (R). In this study, a conductivity meter was utilized to detect the concentration of feed and permeate solutions, and the R was calculated according to equation S2:

$$R(\%)=(1-\frac{C_p}{C_f})\times 100(\%) \quad \text{(S2)}$$

where, C_f and C_p are the conductivity of the feed solution and the permeate, respectively.

The Mg^{2+}/Li^{+} separation performance of the composite membrane was evaluated by simulating brine. A mixed solution of $MgCl_2$ and LiCl (total salinity of 2 g/L) was prepared with a Mg^{2+}/Li^{+} mass ratio of 20. The ion concentrations in the solution before and after permeation were determined by an inductively coupled plasma optical emission spectrometer (ICP-OES). The Mg^{2+}/Li^{+} selectivity of the composite membrane was evaluated using the separation factor $S_{\mathrm{Li,Mg}}$ as shown in equation S3:

$$S_{Li,Mg}=\frac{C_{Li,p}/C_{Mg,p}}{C_{Li,f}/C_{Mg,f}} \quad \text{(S3)}$$

where, $C_{Li,f}$ and $C_{Li,p}$ represent the concentration of lithium ions in the solution before and after infiltration, respectively; Similarly, $C_{Mg,f}$ and $C_{Mg,p}$ represent the concentration of magnesium ions in the solution before and after infiltration, respectively.

2. Supplementary Figures

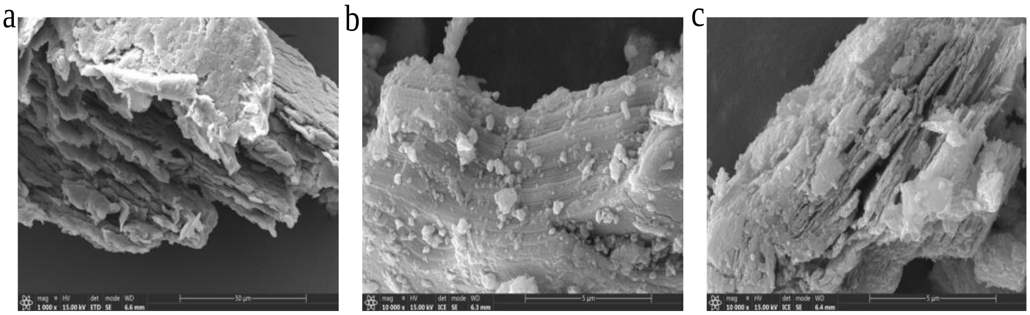

Fig. S1 (a-c) SEM images of GO nanosheets, MAX phase, and MXene nanosheets.

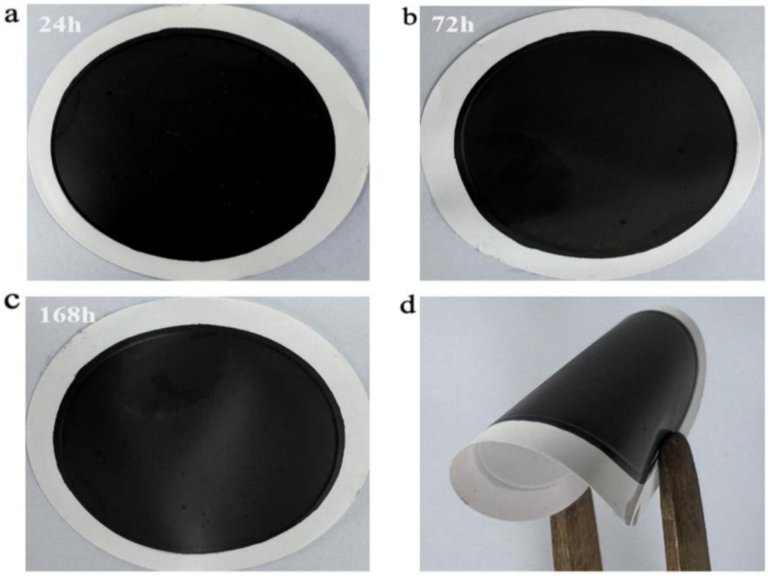

Fig. S2 Digital images of M3 after different immersion times in salt lake brine: (a) 24 h, (b) 72 h, (c) 168 h; (d) bending picture of M3.

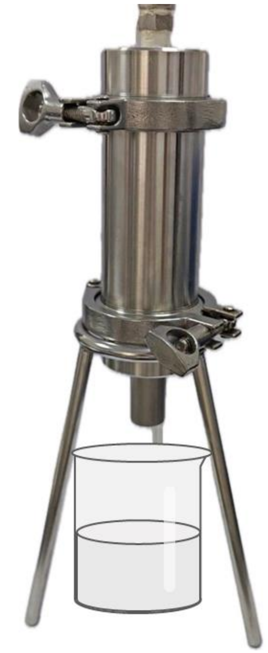

Fig. S3 The dead-end filtration device.

3. Supplementary Tables

Table S1 Composition of different composite membranes.

Membrane	GO (mg)	MXene (mg)	PEI solution (wt%)
M0	1.5	1	0
M1	1.5	1	0.5
M2	1.5	1	0.75
M3	1.5	1	1.0
M4	1.5	1	1.25
M5	1.5	1	1.5

Table S2 Actual salt lake brine ion concentration in Tibet, China.

**Ion**	**Na^+**	**Li^+**	**K^+**	**Ca^{2+}**	**Mg^{2+}**	**Cl^-**	**SO_4^{2-}**	**CO_3^{2-}**	**$B_4O_7^{2-}$**
Concentration（ppm）	37500	840	6335	294	3765	60062	14097	697	688
